# Chlorogenic Acid Promotes Autophagy and Alleviates *Salmonella* Typhimurium Infection Through the lncRNAGAS5/miR-23a/PTEN Axis and the p38 MAPK Pathway

**DOI:** 10.3389/fcell.2020.552020

**Published:** 2020-11-05

**Authors:** Shirui Tan, Fang Yan, Qingrong Li, Yaping Liang, Junxu Yu, Zhenjun Li, Feifei He, Rongpeng Li, Ming Li

**Affiliations:** ^1^Center of Life Sciences, School of Life Sciences, Yunnan University, Kunming, China; ^2^The Second Affiliated Hospital of Kunming Medical University, Kunming, China; ^3^Pneumology Department, Suzhou Kowloon Hospital, School of Medicine, Shanghai Jiao Tong University, Suzhou, China; ^4^School of Agriculture, Yunnan University, Kunming, China; ^5^Key Laboratory of Biotechnology for Medicinal Plants of Jiangsu Province and School of Life Sciences, Jiangsu Normal University, Xuzhou, China

**Keywords:** chlorogenic acid, *Salmonella typhimurium*, lncRNAGAS5, microRNA-23a, PTEN, p38 MAPK, autophagy

## Abstract

**Background:**

*Salmonella typhimurium (ST*) causes several intestinal diseases. Polyphenols including chlorogenic acid (CGA) inhibit pathogenesis.

**Objective:**

This study aimed to investigate the mechanisms of CGA in *ST* infection.

**Methods:**

The intestinal pathological changes and survival rate of ST-infected mice were measured to verify the protection of CGA on *ST* infection. The antibacterial effects of CGA *in vitro* on the invasion to intestinal epithelial cells and autophagy was evaluated. The relationships among GAS5, miR-23a, and PTEN were verified. Expression of inflammation- and autophagy-related proteins was detected.

**Results:**

CGA treatment alleviated pathological damage, improved the secretion disturbance of intestinal cytokines caused by *ST* infection, and reduced the mortality of mice. Intestinal GAS5 was upregulated after CGA treatment. LncRNA GAS5 competitively bound to miR-23a to upregulate PTEN and inhibit the p38 MAPK pathway. CGA regulated the p38 MAPK pathway through lncRNA GAS5/miR-23a/PTEN axis to promote autophagy in *ST* infection. The functional rescue experiments of miR-23a and PTEN further identified these effects.

**Conclusion:**

CGA promotes autophagy and inhibits *ST* infection through the GAS5/miR-23a/PTEN axis and the p38 MAPK pathway.

## Introduction

*Salmonella* enterica serovar Typhimurium (*S*. Typhimurium, ST) is a Gram-negative facultative intracellular pathogen in humans and animals (Viswanathan et al., 2009; Jiang et al., 2018). The *ST* invades intestinal phagocytic and epithelial cells to achieve its pathogenicity, intracellular replication, and dissemination to other tissues (Birhanu et al., 2018). *ST* is the leading cause of bacterial gastrointestinal infections, enterocolitis, typhoid-like diseases, and severe systemic diseases in human and livestock around the world via oral intake of contamination (Velge et al., 2012). The systemic dissemination of *ST* may cause acute intestinal inflammation, diarrhea and immunodeficiency disorders in people and result in an enormous economic loss in animal husbandry and food industry (Park et al., 2017; Yang et al., 2019). Despite increasing awareness of hygiene and health issues, *ST* infection has increased worldwide and its underlying mechanisms remain unclear (Jain et al., 2018). Therefore, a comprehensive understanding of the mechanisms and new therapeutic agents will provide a novel insight to clarify *ST* infections and develop proper therapies to minimize economic loss.

Antibacterial activities of some agents against a multidrug-resistant strain of *ST* show suppressive actions *in vitro* and in mice (Kim et al., 2018; Mortazavi and Aliakbarlu, 2019). As one of the most abundant polyphenols in human diet, chlorogenic acid (CGA) is mainly from coffee, fruits and vegetables, and is also an important bioactive component in some medicine (Upadhyay and Mohan Rao, 2013). Polyphenols including CGA can inhibit pathogenic bacteria and help regulate intestinal microorganisms (Bao et al., 2019). Studies have shown that CGA has anti-oxidative, anti-inflammatory and antibacterial effects, and improves immune regulation (Tosovic et al., 2017; Zhang et al., 2018; Wang et al., 2019). Importantly, the antibacterial activity of CAG against Salmonella Enteritidis S1 has been reported (Sun et al., 2020). Moreover, *Salmonella* expresses hundreds of small regulatory RNAs, many of which are activated under defined stress and virulence conditions, suggesting a role during host infection (Westermann et al., 2016). Mammalian cells express many long non-coding RNA (lncRNA), which are associated with dysregulation of immunity and host defense (Carpenter and Fitzgerald, 2018). *ST* infection usually causes cell inflammation, and many lncRNAs are differentially expressed in inflammatory diseases. GAS5 is abnormally expressed in autoimmune and inflammatory diseases, especially in patients with bacterial sepsis (Mayama et al., 2016). In some inflammatory diseases, GAS5 affects cell inflammatory responses by regulating TLR4 receptor (Zhao et al., 2020). TLR family including TLR4 is an important cell surface receptor in the process of *ST* infection (Kamble et al., 2017; Muneta et al., 2018; Lin H.H. et al., 2020). However, less attention has been paid to the involvement of lncRNA GAS5 in the molecular mechanism of *ST* infection. In addition, the interaction between CGA and lncRNA has been documented recently, which relieves oxidative stress injury in retinal ganglion cells by upregulation of lncRNA TUG1 expression (Gong et al., 2019). In our preliminary experiments for screening lncRNAs in mice, lncRNA GAS5 expression was remarkably changed after CGA treatment. Hence, we focused on lncRNA GAS5 and tried to figure out the regulatory mechanism of CGA in the treatment of *ST* infections.

In light of the aforementioned information, we hypothesized that CGA upregulates lncRNA GAS5 expression in the model of ST infection. We conducted histochemical and molecular experiments to verify the hypothesis, with the purpose of providing antibacterial agents against *ST* infection.

## Materials and Methods

### Ethics Statement

This study was ratified and supervised by the ethics committee of Yunnan University (Approval number:Yuncae2020334). Significant efforts were made to minimize the animals used and their suffering.

### Animal Grouping and Treatments

After normal feeding for 1 week, 6–8-weeks-old specific pathogen free female C57BL/6 mice (purchased from Changchun Yisi Experimental Animal Technology Co., Ltd., Changchun, China) were grouped into: (1) saline group (*n* = 20, gavaged with 100 μL of normal saline every day for 10 days); (2) Sty group (*n* = 20, gavaged with 100 μL of normal saline every day for 7 days, and given oral ST (5.19 × 10^9^cfu mL^–1^day^–1^p.o) on days 8–10 (Dong et al., 2020); (3) treatment groups of different concentrations of CGA (60 mice were assigned into three groups with 20 mice in each group, gavaged with 10, 60, and 120 mg/kg/day of CGA (Sigma, St. Louis, MO, United States), respectively, and given oral *ST* (5.19 × 10^9^cfu mL^–1^day^–1^p.o (*n* = 20) on days 8–10 after CGA treatment; (4) ST + CGA + kd-NC group (*n* = 20, gavaged with 60 mg/kg/day CGA daily, given oral *ST* (5.19 × 10^9^cfu mL^–1^day^–1^p.o) on days 8–10), and injected with 100 μL of empty vector in the tail vein on days 5 and 10); (5) ST + CGA + kd-GAS5 group (*n* = 20, the rest operations were the same as those in the kd-NC group, except that the tail vein was injected with 100 μL 7.8 × 10^7^ IFUs GAS5 interfering vector) on the 5th day and the 10th day). Gas5 interference vector kd-GAS5, control kd-NC, and sh-NC and sh-GAS5 used in the following experiments were constructed and synthesized by Shanghai Genechem Co., Ltd. (Shanghai, China).

On the 11th day of CGA gavage, the mesenteric lymph nodes, liver, and spleen of 5 mice in each group were aseptically collected for determining of organ indices and bacterial loads of *Salmonella*. Hematoxylin and eosin (HE) staining was used to detect intestinal pathological changes. Another five mice in each group were used for enzyme-linked immunosorbent assay (ELISA) to detect the intestinal cytokine secretion level. The remaining 10 mice in each group were used for the observation of the living situation. The body weight, general state and mortality rate of mice was monitored and recorded.

### HE Staining

The contents of the cecum and colorectum were rinsed with sterile phosphate buffer saline (PBS), and 1 cm of the intestine was cut and fixed in 4% (m/v) paraformaldehyde. After paraffin embedding, the sections at 4 μm were dewaxed and dehydrated, and stained with HE (Beyotime Biotechnology Co., Ltd., Shanghai, China), followed by observation under a microscopy.

### Detection of Bacterial Load of *Salmonella* in Organs

Spleen, liver, cecum, colorectal samples of mice were isolated and weighed. According to organ weight/cadaveric weight, the organ index was calculated. The weighed spleen and liver were ground in pre-cooled PBS, and 100 μL of diluted sample was spread on Luria-Bertani agar plates with 20 mg/mL streptomycin sulfate (Shandong Lukang Pharmaceutical Co., Ltd., Jining, China) at 37°C overnight, for the observation and counting of colonies.

### ELISA

The contents of the cecum after PBS washes were ground using liquid nitrogen, and centrifuged at 3,500 r/min, 4°C for 20 min. The supernatant was collected to detect the levels of nitric oxide (NO), interleukin (IL)-12 (IL-12), and IL-10 secreted in the tissue, or the levels of IL-4, IL-6, tumor necrosis factor-α (TNF-α), and IL-1β in the cells after fixing. The detection protocol was in strict accordance with the instructions of the ELISA kits (Shanghai Lengton Bioscience Co., Ltd, Shanghai, China). In short, the ground tissue supernatant was added into the 96-well ELISA plate, and incubated at 37°C for 2 h after adding the coating solution, and then incubated overnight at 4°C. After washing in PBS containing 0.05% (v/v) Tween-20 repeatedly, the sample was blocked using 10% calf serum overnight at 4°C. After washing, the primary antibody was added and incubated at 37°C for 2 h, and the second antibody was incubated at the same temperature for 1.5 h. Then the color reaction was carried out. The termination solution was added to terminate the reaction, and the value was recorded on the microplate.

### Cytotoxicity Test

CGA diluted with high-glucose Dulbecco’s modified Eagle’s medium (DMEM) was added to the mouse intestinal epithelial cells MODE-K (American Type Culture Collection, Manassas, VA, United States) cultured in 96-well microplates, so that the final concentration of CGA was 0, 10, 40, 80, and 160 μg/mL, with 3 replicates for each concentration. The microplates treated with CGA were placed in a 37°C incubator and cultured for 5 h with 5% CO_2_. Afterward, the microplates were centrifuged at 1,000 r/min for 10 min. From each well, 100 μL of supernatant was aspirated and placed into new 96-well microplates. Each well was incubated with 100 μL of prepared lactate dehydrogenase (LDH) reagent for 15 min in the dark. Then absorbance at the wavelength of 490 nm was measured with a microplate reader (Shanghai Peiou Analytical Instrument Co., Ltd., Shanghai, China) to calculate the amount of LDH released into the supernatant of each well.

### Gentamicin Protection Assay

*ST* cultured overnight was expanded in a new tryptic soy broth (TSB) tube at 1:20. CGA solution was added to make the concentrations at 0, 10, 20, 30, and 40 μg/mL, and cultured at 37°C for 4 h. Then the bacterial suspension was diluted with high-glucose DMEM, so that the ratio of the bacterial counts to the cells (multiplicity of infection value) was 200:1. The suspension was cultured at 37°C for 24 h. The bacterial suspension and MODE-K cells were mixed, centrifuged at 1,000 r/min for 10 min, and then cultured at 37°C for 50 min, so that the bacteria repeatedly invaded MODE-K cells. Next, 1 mL of gentamicin diluted with high-glucose DMEM (with the final concentration being 100 μg/mL) was added into each well and incubated at 37°C for 1 h. After that, 1 mL of 0.2% (m/v) saponin was added into each well to fully lyse the cells. The lysate was diluted at proper ratios, and spread onto the prepared TSB culture plates, which was then placed into a 37°C incubator for 24 h. The number of colonies was counted, and the invasion rate of *ST* treated with CGA was calculated, with the invasion rate of *ST* in the group without CGA treatment as 100%. Subsequently, CGA at the optimal concentration against ST was cocultured with MODE-K cells and assigned into: ST + CGA + NC group (cells were treated with 40 μg/mL CGA, and transfected with GAS5 empty vector), ST + CGA + sh-GAS5 group (cells were treated with 40 μg/mL CGA, and transfected with 20 nM GAS5 interference vector), ST + CGA + sh-GAS5 + in-miR-23a group (the treatment protocol was the same as ST + CGA + sh-GAS5 group, except that miR-23a inhibitor was transfected). The transfection was performed in line with the instructions of Lipofectamine^TM^ 2000 (Invitrogen, Carlsbad, CA, United States).

### Reverse Transcription Quantitative Polymerase Chain Reaction (RT-qPCR)

Total RNA in the tissue and cells was extracted using RNAiso Plus (TAKARA, Otsu, Shiga, Japan) and Trizol LS Reagent (TAKARA), respectively. Subsequently, the reliability of the obtained RNA was verified using denaturing formaldehyde agarose gel electrophoresis. Reverse transcription was performed using the PrimeScript RT kit (TAKARA). Standard real-time qPCR was performed according to the instructions of SYBR Premix Ex Taq (TAKARA), and miR and mRNA levels were quantified according to the 2^–ΔΔCt^ method. U6 or glyceraldehyde-3-phosphate dehydrogenase (GAPDH) was used as the control. All primers were synthesized by Sangon Biotech, Shanghai, China. The primer sequences are shown in Table 1.

**TABLE 1:** Primer sequences of RT-qPCR.

Gene	Sequence (5′–3′)
GAS5	F: CTTCTGGGCTCAAGTGATCCT
	R: TTGTGCCATGAGACTCCATCAG
miR-23a	F: AATCCCTGGCAATGTGAT
	R: GTGTAACACGTCTATACGCCCA
U6	F: CTCGCTTCGGCAGCACA
	R: GTGTCGTGGAGTCGGCAA
PTEN	F: GAAGTATGACAAAGATGA
	R: AGAGCTTCTTGGAGACTG
IL-1β	F: ATAAGCCCACTCTACACCTCTGA
	R: ATTGGCCCTGAAAGGAGAGAGA
IL-6	F: GCTTCTTAGCGCTAGCCTCAATG
	R: TGG GGCTGATTGGAAACCTTATT
IL-4	F: TGCTGCCTCCAAGAACACAACTG
	R: CATGATCGTCTTTAGCCTTTCCA
TNF-α	F: TCC TGCATCCTGTCTGGAAG
	R: 5’-GTCTTCTGGGCCACTGACTG
GAPDH	F: ACCAGGTATCTGCTGGTTG
	R: TAACCATGATGTCAGCGTGGT

*GAS5, growth arrest-specific 5; IL, interleukin; PTEN, gene of phosphate and tension homology deleted on chromsome ten; RT-qPCR, reverse transcription quantitative polymerase chain reaction; GAPDH, glyceraldehyde-3-phosphate dehydrogenase; miR, microRNA; F, forward; R, reverse.*

### Western Blot Analysis

The cells and cecum tissues of the above groups and mice were ground and homogenized and added with lysate, and lysed with ultrasound. After that, the samples were centrifuged for 10 min. A small amount of sample was taken to determine the protein concentration using bicinchoninic acid (BCA) protein quantification kit (Thermo Fisher Scientific Inc., Waltham, MA, United States) on the microplate reader. For each sample, 50 μg protein was loaded onto a 10–15% sodium dodecyl sulfate-polyacrylamide gel electrophoresis gel with tris-glycine running buffer, and transferred to polyvinylidene fluoride (PVDF) membranes, which was blocked with 5% (m/v) skimm milk or bovine serum albumin (BSA) for 2 h. The membranes were incubated with primary antibodies (Table 2, all from Abcam Inc., Cambridge, MA, United States) at 4°C overnight. The next day after washing with tris-buffered saline with Tween (TBST), the membranes were incubated with goat anti-mouse secondary antibody (1:2,000, ab7063, Abcam) for 2 h. After visualization using enhanced chemiluminescence, the membranes were imaged using the gel imaging system. The band intensity was quantified with Image-Pro Plus 6.0 software (Olympus Optical Co., Ltd, Tokyo, Japan).

**TABLE 2:** Antibodies used in Western blot analysis.

Antibodies	Cat. No. and source	Dilution ratio
P62	ab109012, abcam	1/10,000
LC3I	ab51520, abcam	1/500
LC3II	ab51520, abcam	1/500
P38	ab170099, abcam	1/500
P-P38	ab47363, abcam	1/500
PTEN	ab32199, abcam	1/10,000
GAPDH	ab181602, abcam	1/10,000

*LC3, light chain 3; PTEN, gene of phosphate and tension homology deleted on chromsome ten; GAPDH, glyceraldehyde-3-phosphate dehydrogenase. All antibodies were purchased from Abcam (Cambridge, MA, United States).*

### Transmission Electron Microscope (TEM) Observation

The cells were collected, washed twice with PBS, and fixed on ice for 30 min by adding pre-cooled 2.5% glutaraldehyde fixative. Then the cells were transferred to eppendorf tubes and kept overnight. Then the samples were subjected to acetone gradient dehydration, resin embedding, ultra-thin microtome for sectioning, and uranium acetate-lead citrate double staining, and observed under a transmission electron microscope (HF5000 Hitachi, Japan) for changes of intracellular organelles and nuclei.

### Monodansylcadaverin (MDC) Fluorescence Staining

Cells were washed twice with pre-cooled PBS, and incubated with 0.05 mmoL/L MDC solution (Shanghai Uteam-Biotech Co., Ltd., Shanghai, China) at 37°C for 30 min. Next, cells were fixed with 4% (m/v) paraformaldehyde for 10 min and washed twice with PBS. After the natural air-drying, the cells were observed and imaged under a fluorescence microscope (Olympus, Japan).

### Nuclear/Cytosol Fractionation Experiment

According to the method of a report (Lin H. et al., 2020) and the instructions of the nuclear/cytosol fractionation kit (Invitrogen), the cells were isolated. Briefly, 10^6^ cells were mixed with 300 μL cell disruption buffer, incubated on ice for 10 min, and centrifuged for 3 min. The supernatant was cytosol, and the precipitate was the nuclei. The supernatant was aspirated into RNase-free tubes and placed on ice to perform RNA extraction immediately. Subsequently, lncRNA GAS5 in the nucleus and cytoplasm was detected using RT-qPCR.

### Fluorescence *in situ* Hybridization (FISH) Assay

The localization of GAS5 was detected in intestinal epithelial cells. The cells were allocated into cell group and control group (the cells were treated only with 4′,6-diamidino-2-phenylindole (DAPI) but no GAS5 probe), and seeded in 24-well microplates with coverslips for 24 h. After diluting the lncRNA-GAS5 FISH probe in the hybridization solution at 1:50, 100 μL the mixture was added to each well and hybridized in a 37°C incubator in the dark overnight. The cells were washed with sodium saline citrate the next day, stained with 10 μL DAPI working solution (10 μg/mL), kept still for 8 min, and washed 3 times with PBS. Finally, the coverslips were sealed, observed and imaged as the kit (Guangzhou RiboBio Co., Ltd., Guangzhou, China) instructed.

### RNA Pull-Down Assay

The binding of GAS5 to miR-23a was detected by RNA pull-down assay. Total RNA of tissue (100 μg) was extracted. For each sample, 500 μg streptavidin magnetic beads were mixed with 200 pmol biotin-labeled miR-23a, and incubated with the extracted RNA for 1 h. Elution buffer was added to collect the RNA complex that had been pulled down. Quantitative analysis of lncRNA GAS5 levels was fulfilled using RT-qPCR. The specific operations were based on the instructions of GENEWIZ (China) and biotin-labeled Magnetic RNA-protein pull-down kit (Thermo Fisher Scientific).

### Dual Luciferase Reporter Gene Assay

The PTEN and GAS5 sequences containing miR-23a binding site were synthesized, respectively, and PTEN and GAS5 wild-type (WT) plasmids (PTEN/GAS5-WT) were constructed. Based on the WT plasmids, the binding sites were mutated to construct PTEN and GAS5 mutant (MUT) plasmids (PTEN/GAS5-MUT). These plasmids were mixed with NC and miR-23a and transfected into 293T cells. Cells were collected and lysed 48 h after transfection, and luciferase activity was detected using a luciferase detection kit (BioVision, San Francisco, CA, United States) and a Glomax20/20 luminometer fluorescence detector (Promega, Madison, Wisconsin, United States).

### Statistical Analysis

Data were analyzed using SPSS 21.0 (IBM Corp. Armonk, NY, United States). Kolmogorov-SmiRnov test showed a normal distribution of the data, and data are expressed as mean ± standard deviation. Comparisons between two groups were performed using independent sample *t*-tests. Comparisons among multiple groups were performed using one-way or two-way analysis of variance (ANOVA). Pairwise comparisons after ANOVA were performed using Tukey’s multiple comparison test. Survival analysis was performed using Kaplan-Meier analysis. The *p*-value was calculated using a two-sided test, and *p* < 0.05 was considered statistically significant.

## Results

### CGA Alleviated Pathological Damage Caused by *ST* Infection

**FIGURE 1:**
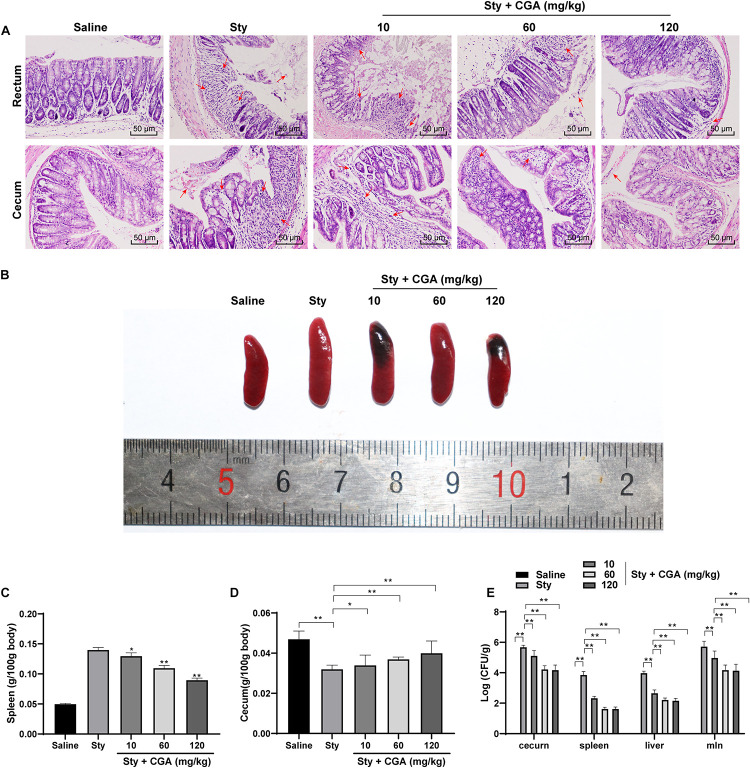
CGA alleviated the pathological damage of *ST*-infected mice. Female C57BL/6 mice were treated with *ST* infection and different concentrations of CGA. **(A)** HE staining detected the pathology of rectum and cecum of mice (×200); the arrow points to the bleeding point; **(B)** spleen morphology of mice in each group; **(C)** spleen organ index of mice in each group; **(D)** cecum organ index of mice in each group; **(E)** *Salmonella* loads in cecum, spleen, liver and mesenteric lymph nodes of mice; *n* = 5. The data in panels C/D were analyzed using one-way ANOVA and Tukey’s multiple comparison test, and data in panel E were analyzed using two-way ANOVA and Tukey’s multiple comparison test, *p < 0.05, **p < 0.01.

To evaluate the effect of CGA on *ST* infection, we established a mouse enteritis model induced by *ST* and treated the mice with different concentrations of CGA by gavage. The pathological damage of the colorectum and cecum of mice was evaluated using HE staining. The results showed that the intestinal rectum gland and cecal gland of control mice were intact; while most of the glands of *ST*-infected mice were destroyed, the intestinal mucosa was shed, with massive inflammatory cell infiltration and bleeding spots. With the increasing concentrations of CGA, the structures of rectal and cecal glands of *ST*-infected mice were intact, and inflammatory cell infiltration and bleeding points were gradually reduced (Figure 1A). The spleen in *ST*-infected mice was enlarged and the weight was notably increased compared with those of control mice. With increasing concentrations of CGA, the spleen swelling caused by *ST* infection was attenuated, and the decreased contents in cecum and decreased weight were gradually improved (Figures 1B–D). In addition, no *Salmonella* was detected in the organs of control mice, while a large amount of *Salmonella* was present in the organs of *ST*-infected mice. Increasing concentrations of CGA significantly reduced the bacterial loads in the spleen, liver and mesenteric lymph nodes of *ST*-infected mice (all *p* < 0.01) (Figure 1E). In summary, CGA alleviated the pathological damage of mice caused by *ST* infection.

### CGA Regulated the Secretion Balance of Cytokines in the Intestinal Tract of *ST*-Infected Mice and Reduced the Mortality

**FIGURE 2:**
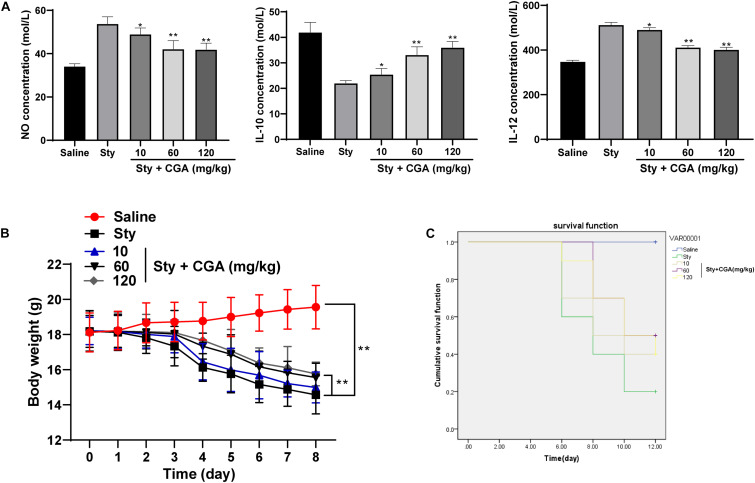
CGA regulates the secretion balance of cytokines in *ST*-infected intestine and alleviates the mortality of mice. *ST*-infected mice were treated with different concentrations of CGA. **(A)** Intestinal cytokine secretion levels of mice in each group measured by ELISA (*n* = 5); **(B)** daily measurement of body weight of mice in each group (*n* = 10); **(C)** survival of mice in each group (*n* = 10). The abscissa is the observation time, and the ordinate is the survival rate. The data in **(A)** were analyzed using one-way ANOVA and Tukey’s multiple comparison test, data in panel B were analyzed using two-way ANOVA and Tukey’s multiple comparison test, and data in panel C were analyzed using Kaplan-Meier analysis; compared with the sty group, *p < 0.05, **p < 0.01.

The levels of NO and IL-12 secretion in the intestinal tissue of *ST*-infected mice were increased, while the level of IL-10 secretion was significantly reduced. These changes were reversed after treatment with different concentrations of CGA (Figure 2A) (all *p* < 0.01). It possibly indicated that CGA might maintain the intestinal immune balance and relieve the intestinal inflammatory responses by regulating the secretion of cytokines. The weight of control mice was increased slowly, and the weight of *ST*-infected mice was lost severely, but different concentrations of CGA alleviated the weight loss (Figure 2B). In addition, no mice died in the control group, while the survival rate of *ST*-infected mice was only 20% on the 12th day, and the survival rate in *ST*-infected mice treated with CGA was increased (Figure 2C). In conclusion, CGA could improve the secretion disturbance of intestinal cytokines caused by *ST* infection, alleviate the weight loss, and reduce the mortality of mice.

### CGA Reduced Intestinal Damage Caused by *ST* by Upregulating lncRNA GAS5 Expression

**FIGURE 3:**
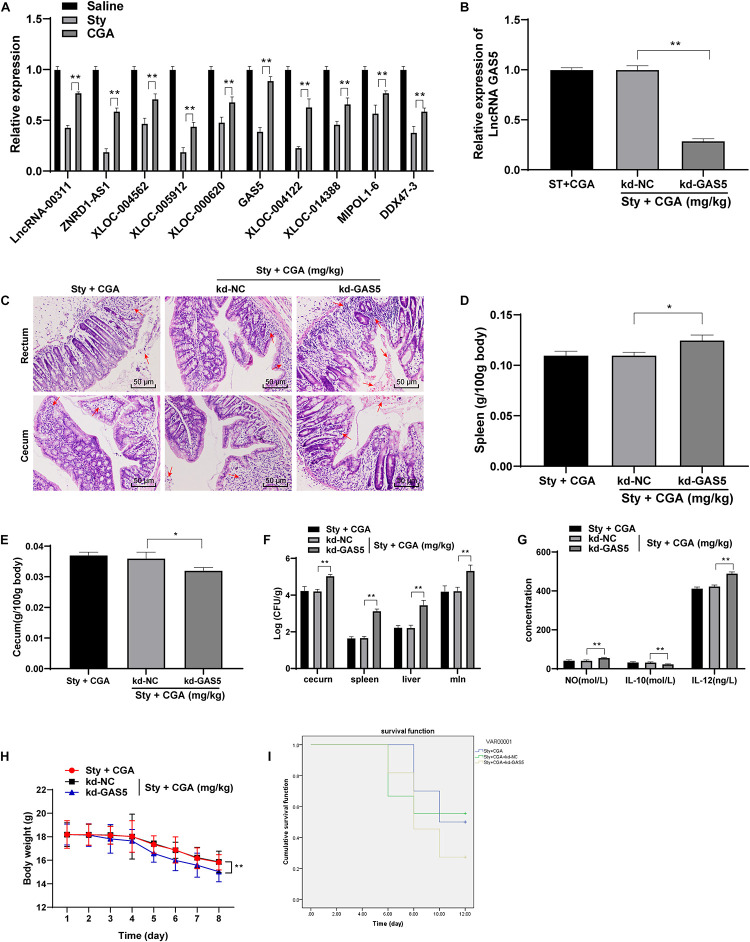
CGA reduced intestinal damage caused by *ST* infection by upregulating lncRNA GAS5 expression. **(A)** RT-qPCR detected differential expression of lncRNAs in intestinal tissue of mice (*n* = 5); **(B)** RT-qPCR detected lncRNA GAS5 expression (*n* = 5); **(C)** HE staining detected pathology of rectum and cecum of mice (×200) (*n* = 5)’ the arrow points to the bleeding point; **(D)** spleen organ index of mice in each group (*n* = 5); **(E)** cecum organ index of mice in each group (*n* = 5); **(F)** *Salmonella* loads in the cecum, spleen, liver and mesenteric lymph nodes of mice (*n* = 5); **(G)** intestinal cytokine secretion level of mice in each group (*n* = 5); **(H)** daily measurement of body weight of mice in each group (*n* = 10); **(I)** survival status of each group (*n* = 10). The abscissa is the observation time, and the ordinate is the survival rate. Kd-GAS5 is the interference vector of GAS5, and the empty vector (kd-NC) is used as the control. The data in **(B,D)** were analyzed using one-way ANOVA and Tukey’s multiple comparison test, data in **(A,F,G,H)** were analyzed using two-way ANOVA and Tukey’s multiple comparison test, and data in **(I)** was analyzed using Kaplan-Meier analysis; *p < 0.05, **p < 0.01.

To understand the protective mechanism of CGA against *ST* infection, we referenced a large amount of publications on immunity and anti-inflammation, and found that lncRNAs play a certain role in these physiological processes. Therefore, we selected 10 related lncRNAs in the recent references and detected the expression in the intestinal tissue of *ST*-infected mice using RT-qPCR. It turned out that GAS5 expression in the intestinal tissue of *ST*-infected mice was decreased significantly, but upregulated significantly in *ST*-infected mice treated with a high concentration of CGA (Figure 3A). GAS5 is abnormally downregulated in patients with bacterial sepsis (Mayama et al., 2016). In some inflammatory diseases, GAS5 affects cell inflammatory responses by regulating TLR4 receptor (Zhao et al., 2020), which is an important cell surface receptor in *ST* infection (Kamble et al., 2017; Muneta et al., 2018; Lin H.H. et al., 2020). Therefore, we speculated that CGA might upregulate GAS5 expression in the intestinal tissue of *ST*-infected mice, thus exerting a protective effect. To confirm this speculation, we injected the GAS5 interference vector (kd-GAS5) into the *ST*-infected mice via the tail vein, which successfully knocked down GAS5 expression (Figure 3B). Then we evaluated the colorectal and cecal pathological damage using HE staining. After GAS5 knockdown, the intestinal glands of mice were destroyed, intestinal mucosa was shed, inflammatory cells infiltrated, and blood spots took place (Figure 3C); the spleen and cecum organ index of mice was increased (Figures 3D,E); the bacterial loads in the spleen, liver and mesenteric lymph nodes of mice were significantly increased (all *p* < 0.01) (Figure 3F). ELISA showed that the levels of NO and IL-12 in the intestinal tissue of *ST*-infected mice were significantly increased, while the IL-10 secretion was reduced after GAS5 knockdown (Figure 3G). Furthermore, the mice lost weight significantly (Figure 3H) and the survival rate was reduced (Figure 3I) after GAS5 knockdown. In summary, GAS5 knockdown could reverse the protective effect of CGA on *ST*-infected intestine.

### CGA Inhibited the Invasion of *ST* to Intestinal Epithelial Cells and Improved the Secretion Balance of Inflammatory Cytokines

**FIGURE 4:**
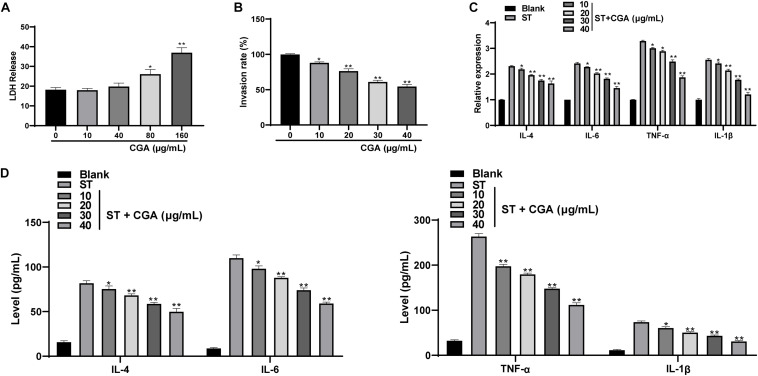
CGA inhibited the proliferation of *ST* in intestinal epithelial cells and regulates the secretion balance of inflammatory factors. *ST*-infected MODE-K cells were treated with different concentrations of CGA. **(A)** cytotoxicity test was performed to detect the cytotoxicity of CGA to MODE-K cells; **(B)** gentamicin protection assay was used to determine the invasion rate of *ST* after different doses of CGA treatments; **(C)** RT-qPCR detected IL-4, IL-6, TNF-α, and IL-1βlevels; **(D)** ELISA detected IL-4, IL-6, TNF-α, and IL-1β levels. All experiments were repeated 3 times. The data in **(A,B)** were analyzed using one-way ANOVA and Tukey’s multiple comparison test, and data in **(C,D)** were analyzed using two-way ANOVA and Tukey’s multiple comparison test; compared with 0 μg/mL CGA (ST) group. *p < 0.05, **p < 0.01.

To investigate whether CGA could inhibit the *in vitro* infection of *ST*, we chose mouse intestinal epithelial cells MODE-K for *in vitro* experiments. First, the cytotoxicity tests found that CGA had no cytotoxicity at the concentrations of 0, 10, and 40 μg/mL, but had weak cytotoxicity at the concentration of 80 μg/mL. The results indicated that when the effective concentration of CGA was from 0 to 40 μg/mL, it did not affect the activity of intestinal epithelial cells (Figure 4A). Then gentamicin protection assay found that when the CGA concentration was 40 μg/mL, the invasion rate of *ST* to MODE-K cells was minimal (Figure 4B). Moreover, RT-qPCR and ELISA showed that compared with those of the control, the levels of various inflammatory factors (IL-4, IL-6, TNF-α, and IL-1β) in *ST*-infected MODE-K cells were increased significantly, and these levels were gradually reduced after treatments with different doses of CGA (Figures 4C,D) (*p* < 0.01).

### Low Expression of lncRNA GAS5 Attenuated the Protective Effect of CGA Against *ST* Invasion to Intestinal Epithelial Cells

**FIGURE 5:**
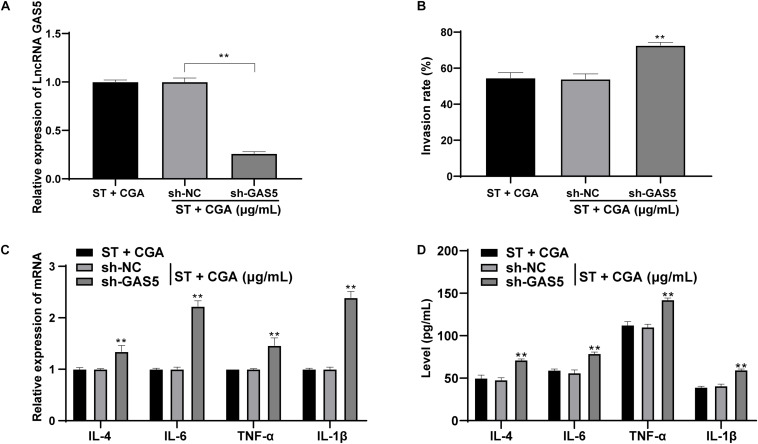
Low expression of lncRNA GAS5 attenuated the protective effect of CGA against *ST* invasion to MODE-K cells. GAS5 expression was knocked down in MODE-K cells, and then the toxic effect of CGA on cells was observed. **(A)** RT-qPCR was used to detect the expression of lncRNA GAS5; **(B)** gentamicin protection assay was used to detect the invasion rate of *ST* to MODE-K cells after different treatments; **(C)** RT-qPCR was used to detect IL-4, IL-6, TNF-α, and IL-1β levels; **(D)** ELISA detected IL-4, IL-6, TNF-α, and IL-1β levels. All experiments were repeated 3 times; The data in **(A,B)** were analyzed using one-way ANOVA and Tukey’s multiple comparison test; data in **(C,D)** were analyzed using two-way ANOVA and Tukey’s multiple comparison test; compared with ST + CGA group, **p < 0.01.

Similarly, to confirm the effect of CGA on *ST* infection was achieved through lncRNA GAS5 *in vitro*, CGA concentration at 40 μg/mL was selected for experiments and low expression of GAS5 was transfected into MODE-K cells (Figure 5A). The gentamicin protection assay found that the bacterial invasion rate to MODE-K cells was increased significantly after GAS5 silencing (*p* < 0.05) (Figure 5B). RT-qPCR and ELISA showed that GAS5 silencing caused significant increases in the levels of inflammatory factors in MODE-K cells (all *p* < 0.05) (Figures 5C,D).

### LncRNA GAS5 Competitively Bound to miR-23a to Upregulate PTEN Expression

**FIGURE 6:**
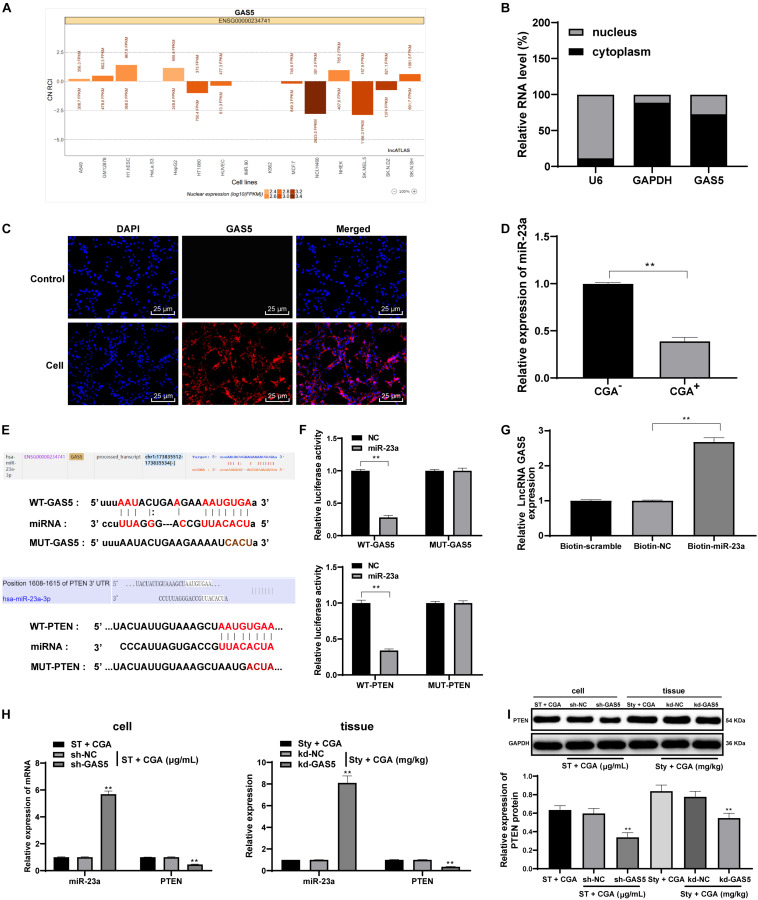
LncRNA GAS5 competitively binds miR-23a to upregulate PTEN expression. The downstream regulatory molecules of GAS5 were analyzed and verified. **(A)** the biological website confirmed that lncRNA GAS5 is located in the cytoplasm; **(B)** nuclear/cytosol fractionation experiments confirmed the subcellular localization of lncRNA; **(C)** FISH assay confirmed that lncRNA GAS5 is localized in the cytoplasm; **(D)** RT-qPCR detection of the expression of mioR-23a in tissue before and after CGA treatment; **(E,F)** dual luciferase gene reporter assay accurately verified the existence of binding regions of lncRNAGAS5 and miR-23a, and miR-23a and PTEN genes; **(G)** RNA pull-down assay found there is a binding region between lncRNA GAS5 and miR-23a; **(H)** RT-qPCR was used to detect the expression of miR-23a and PTEN; **(I)** Western blot was used to detect the protein level of PTEN. All the experiments were repeated 3 times. The data in **(G,I)** were analyzed using one-way ANOVA and Tukey’s multiple comparison test; the data in **(B,F,H)** were analyzed using two-way ANOVA and Tukey’s multiple comparison test; and the data in **(D)** were analyzed using independent *t*-test; compared with sh-NC or kd-NC groups, **p < 0.01.

From the above results *in vivo* and *in vitro*, we confirmed that CGA exerts a protective effect against *ST* infection by upregulating the expression of GAS5. Recently, the competing endogenous RNA (ceRNA) regulation model describes lncRNA as a sponge for miR to indirectly regulate miR downstream target genes in many disease processes (Han et al., 2020). The ceRNA network of GAS5 has been reported (Liu et al., 2019; Fang et al., 2020). However, we did not know the downstream mechanisms of GAS5. Actually, the activities of lncRNA depend on its subcellular localization. For this purpose, we checked through biological websites and found that GAS5 is partially located in the cytoplasm (Figure 6A). Furthermore, nuclear/cytosol fractionation experiments (Figure 6B), FISH assay (Figure 6C) and dual-luciferase reporter gene assay (Figure 6F) confirmed that lncRNA GAS5 was localized in the cytosol in intestinal epithelial cells. GAS5 can play a role in regulating diseases through the ceRNA mechanism. Through literature search, we found that there were many abnormal expression of miR in mesenteric lymph nodes after *ST* infection (Herrera-Uribe et al., 2018). Therefore, we screened these miRs in the biological websites, and found miR-23a has binding relation to GAS5 (Figure 6E). Then we detected the expression of miR-23a in intestinal tissues of *ST*-infected mice before and after CGA treatment by RT-qPCR. The results showed that the expression of miR-23a in intestinal tissues of *ST*-infected mice treated with CGA was significantly downregulated (Figure 6D). Therefore, we speculated that GAS5 may protect against ST infection by competitively binding to miR-23a. To confirm this speculation, we found that there was a binding region between GAS5 and miR-23a through RNA pull-down assay and dual-luciferase reporter gene assay (Figures 6F,G). It has been reported that PTEN gene deletion can enhance the sensitivity of mice to *ST* infection (Howe et al., 2019). In addition, we predicted through the biological websites that miR-23a has multiple target genes, including PTEN. Then we verified the binding regions of miR-23a and PTEN genes by dual-luciferase reporter gene assay (Figures 6E,F). After GAS5 knockdown, miR-23a was upregulated and PTEN levels were significantly downregulated (Figures 6H,I) (all *p* < 0.01).

### Low Expression of miR-23a Reversed the Promoting Effect of Low Expression of GAS5 on *ST* Invasion to Intestinal Epithelial Cells

**FIGURE 7:**
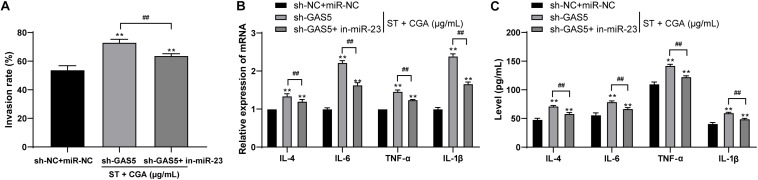
Low expression of miR-23a reversed the promoting effect of low expression of lncRNA GAS5 on the invasion to intestinal epithelial cells by *ST*. *ST*-infected MODE-K cells lowly expressing GAS5 were treated with low miR-23a expression to observe the protective effect of CGA on the cells. **(A)** Gentamicin protection assay was used to detect the invasion rate of *ST* after different CGA treatments; **(B)** RT-qPCR was used to detect the mRNA levels of IL-4, IL-6, TNF-α, and IL-1β; **(C)** ELISA was used to detect the levels of IL-4, IL-6, TNF-α, and IL-1β. All the experiments were repeated 3 times. The data in **(A,B)** were analyzed using *t*-test, and data in **(C)** were analyzed using two-way ANOVA and Tukey’s multiple comparison test. Compared with sh-GAS5 group, **p < 0.01, ^##^*p* < 0.01.

To confirm that GAS5 played a protective role against *ST* infection through competitive binding, we performed cell rescue experiments. MODE-K cells poorly expressing GAS5 were transfected with miR-23a inhibitor. Then we measured the bacterial invasion rate to the intestinal epithelial cells using gentamicin protection assay. Compared with those of GAS5 silencing alone, the bacterial invasion rate to MODE-K cells was decreased significantly (Figure 7A), and the levels of IL-4, IL-6, TNF-α, and IL-1β in the MODE-K cells were reduced after combined treatment of sh-GAS5 + in-miR-23a (Figures 7B,C) (all *p* < 0.05).

### CGA Regulated the p38 MAPK Pathway Through the GAS5/miR-23a/PTEN Axis to Promote Autophagy in *ST* Infection

**FIGURE 8:**
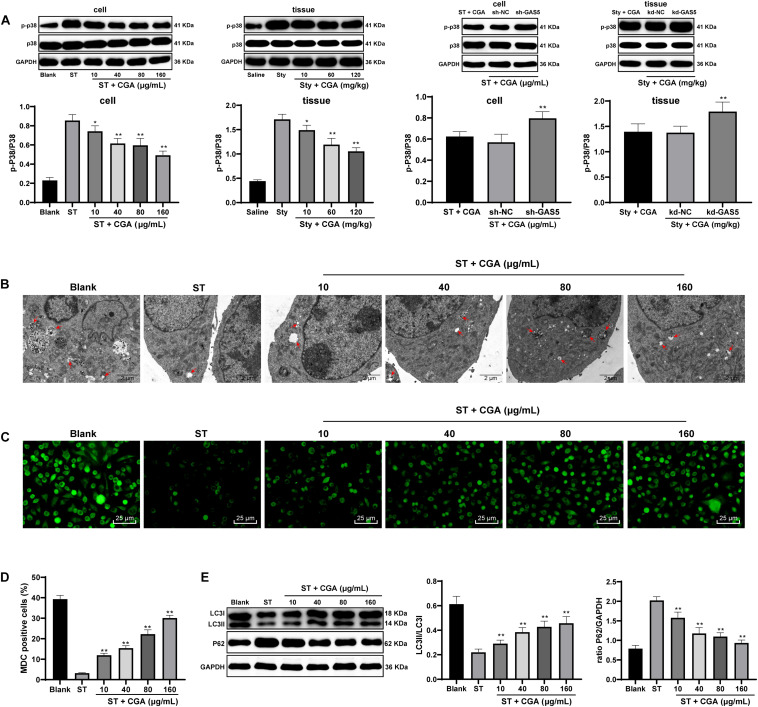
CGA regulated the p38 MAPK signaling pathway through the lncRNAGAS5/miR-23a/PTEN axis to promote autophagy in *ST* infection. Effect of different concentrations of CGA on autophagy of MODE-K cells infected by *ST* was discussed. **(A)** Western blot analysis was used to detect the expression of p38 MAPK signaling pathway-related proteins in tissue and cells; **(B)** autophagosomes and colonies were observed by TEM (×5,000); **(C,D)** MDC fluorescence staining was used to detect cellular autophagy in each group; **(D)** the relative expression in MDC-positive cells; **(E)** Western blot detected the expression of autophagy-related proteins P62 and LC3II/LC3I. All the experiments were repeated 3 times. The data in **(A,D,E)** were analyzed using one-way ANOVA and Tukey’s multiple comparison test. Compared with Blank group, ST + CGA group or Sty + CGA group, *p < 0.05, **p < 0.01.

To explore the downstream pathways regulated by PTEN, Western blot analysis was used to detect the expression of p38MAPK pathway-related proteins in intestinal tissue and cells. CGA treatment reduced the activation level of p38 MAPK pathway, and knocking down GAS5 partially reversed the inhibitory effect of CGA on the p38 MAPK pathway (Figure 8A). In addition, autophagy plays an important role in *ST* infection, and moderate activation of autophagy is beneficial to inhibit the proliferation of *Salmonella* (Lv et al., 2012). It triggered us to speculate that the effect of CGA on ST infection may relate to autophagy. To confirm this hypothesis, the autophagy of intestinal epithelial cells after CGA treatment was detected by TEM, MDC fluorescence staining and immunoblotting. With increasing CGA concentrations, the autophagic vesicles and intracellular bacteria with bilayer membrane structure were increased, and the intracellular fluorescence distribution intensity and LC3II/LC3I were increased, and P62 gradually was decreased (Figures 8B–E). It can be seen that CGA treatment promoted the autophagy of intestinal epithelial cells.

### PTEN Knockdown Inhibited Autophagy and Reverses the Protective Effect of CGA Against *ST* Infection

**FIGURE 9:**
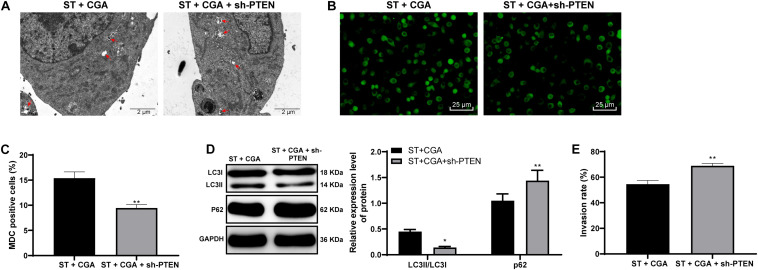
PTEN knockdown inhibited autophagy and reversed the protective effect of CGA on *ST* infection. The autophagy level of MODE-K cells was observed after PTEN expression was knocked down. **(A)** TEM was used to observe the autophagosomes and colonies of cells in each group (×5,000); **(B)** MDC fluorescence staining was used to detect the cellular autophagy in each group; **(C)** the relative expression of MDC-positive cells; **(D)** western blot detection for quantification of the expression of autophagy-related proteins; **(E)** gentamicin protection assay was used to detect the invasion rate of *ST* to MODE-K cells after different CGA treatments. All the experiments were repeated 3 times. The data in **(A,D)** were analyzed using *t*-test for statistical analysis, and data in panel E were analyzed using two-way ANOVA and Tukey’s multiple comparison test. Compared with ST + CGA group, *p < 0.05, **p < 0.01.

To confirm that CGA regulates the autophagy through the lncRNA GAS5/miR-23a/PTEN axis, we performed an *in vitro* functional rescue experiment. Cells were assigned into CGA group and the CGA + sh-PTEN group. TEM, MDC fluorescence staining and immunoblotting were used to detect the autophagy of MODE-K cells, and the gentamicin protection assay was used to determine the invasion rate of *ST* to MODE-K cells. After the combined treatment with CGA and PTEN knockdown, the autophagy of MODE-K cells infected by *ST* was inhibited, and the invasion rate of *ST* to MODE-K cells was increased significantly (all *p* < 0.05) (Figures 9A–E). It is suggested that the promotion of autophagy by CGA is realized through the GAS5/miR-23a/PTEN axis.

## Discussion

### Beneficial Role of CGA in ST Infection

CGA has antibacterial activities against Gram-negative and Gram-positive bacteria (He et al., 2019). RNAseq profiling of various cell types infected with *Salmonella* has provided a treasure trove of lncRNAs that are regulated upon immune cell stimulation or pathogen invasion, but their molecular functions remain uncharacterized (Garcia-Del Portillo and Pucciarelli, 2017; Yu et al., 2018). But the sole or combined roles of CGA and lncRNA GAS5 in *ST* infection are incompletely understood. In this study, we verified the hypothesis that CGA upregulates lncRNA GAS5 in epithelial cells to promote autophagy in the model of *ST* infection via the miR-23a/PTEN/p38MAPK axis.

CGA has certain therapeutic effects on ulcerative colitis, inhibits intestinal inflammation, weight loss, diarrhea, and improves the immune regulation of intestinal microbes (Zhang et al., 2017). In this experiment, with the increase of CGA concentrations, the structures of rectal and cecal glands of *ST*-infected mice were intact, the inflammatory cell infiltration and bleeding points were decreased gradually, the spleen swelling was attenuated, and bacterial load was decreased significantly. The incorporation of cadmium selenide and CGA into nanoparticles can destroy the cell wall of bacteria and inhibit the proliferation of bacteria, which is probably due to the increase of active oxygen caused by the release of CGA (Wang et al., 2020). Briefly, CGA could improve pathological damage caused by *ST* infection. During the process of inflammation, the secretion of IL-12 is related to the clearance of bacteria by macrophages (Yang et al., 2018). Moreover, after oral administration of CGA, NO, and IL-12 levels were decreased, IL-10 was elevated, and body weight loss and low survival rate of *ST*-infected mice were alleviated. In methicillin-resistant *Staphylococcus aureus*-infected mice, the levels of IL-6, TNF-α and IL-10 are upregulated, and bacterial load in spleen, liver and kidney is surged (Li et al., 2020). CGA inhibits the secretion of IL-2, IFN-g, and IL-12 but facilitates IL-4 and IL-10 with dose dependence (Chauhan et al., 2012). In summary, CGA regulates the balance of cytokines in the intestinal tract and reduces the mortality of *ST*-infected mice. Moreover, to investigate whether CGA could inhibit the *in vitro ST* infection, we chose mouse intestinal epithelial cells MODE-K for *in vitro* experiments. The results revealed that the levels of inflammatory factors (IL-4, IL-6, TNF-α, and IL-1β) in *ST*-infected MODE-K cells were gradually reduced after treatments with different doses of CGA. Consistently, CGA alleviates renal injury by reducing inflammation and inducing epithelial cells proliferation (Arfian et al., 2019). CGA abrogates the upregulation of TNF-α and IL-1β and histological changes induced by NaAsO treatment (Dkhil et al., 2020). Taken together, CGA treatment can alleviate *ST* infection *in vivo* and *in vitro*.

### GAS5 Knockdown in the Protective Effect of CGA on the *ST*-Infected Mice and MODE-K Cells

*ST* infection impacts nuclear RNA decay, which in turn drives the accumulation of unstable nuclear lncRNAs, some of which may have protective effects against this common bacterial pathogen (Koops et al., 2018). After reference retrieval, we focused on GAS5. GAS5 expression was decreased in the intestinal tissue of *ST*-infected mice, but increased after administration of CGA. GAS5 levels are alerted in virus or bacterial infectious diseases. GAS5 is important in immune functions and pathogenesis of inflammatory and infectious diseases (Mayama et al., 2016). GAS5 monitors cell inflammatory responses by regulating TLR4 receptor (Zhao et al., 2020), which is important in *ST* infection (Kamble et al., 2017; Muneta et al., 2018; Lin H.H. et al., 2020). After GAS5 knockdown, the intestinal gland of mice was destroyed, inflammatory cells infiltrated, the spleen and cecum organ index of mice was increased, bacteria were increased, the secretion levels of NO and IL-12 were increased, while IL-10 and the survival rate were decreased. The strain deficient of bacterial sialidase is more easily cleared by reducing the inhibition of GAS5, inducing more IL-12 in macrophages (Yang et al., 2018). *In vivo*, low expression of GAS5 was transfected into MODE-K cells. After GAS5 knockdown, the invasion rate of bacteria into MODE-K cells was increased, and the levels of IL-4, IL-6, TNF-α, and IL-1β increased. GAS5 overexpression inhibited TNF-α, IL-6, and IL-1β, oxidative stress and pyroptosis of high-glucose-induced renal tubular cells (Xie et al., 2019). In conclusion, GAS5 knockdown could reverse the protective effect of CGA on the *ST*-infected mice and MODE-K cells.

### The ceRNA Network of GAS5/miR-23a/PTEN

A recent study indicates that mRNAs that co-express with lncRNAs are mainly involved in regulating *ST* infection (Yu et al., 2018). Then our focus shifted to the downstream mechanism of lncRNA GAS5 involving in the protective effect of CGA against *ST* infection. LncRNA GAS5 was located in the cytoplasm of intestinal epithelial cells, suggesting that GAS5 may play a regulatory role in diseases through the ceRNA network. The ceRNA network of GAS5 has been reported (Liu et al., 2019; Fang et al., 2020). After literature search, we found that there were many abnormal expression of miR in mesenteric lymph nodes after ST infection (Herrera-Uribe et al., 2018). Additionally, biological website prediction, RNA pull-down and dual luciferase reporter gene assays confirmed that miR-23a has binding relation to GAS5. The expression of miR-23a in intestinal tissues of *ST*-infected mice treated with CGA was significantly downregulated. Additionally, PTEN gene deletion can enhance the sensitivity of mice to *ST* infection (Howe et al., 2019). miR-23a has multiple target genes, including PTEN. Then we verified the binding regions of miR-23a and PTEN genes by dual-luciferase reporter gene assay. After GAS5 knockdown, miR-23a was upregulated and PTEN levels were significantly downregulated. Intriguingly, lncRNA GAS5 acts as a sponge platform to competitively decrease miR-23a expression, and miR-23a degrading PTEN further influenced the downstream pathway PI3K/Akt/mTOR/Snail in hepatic fibrosis (Dong et al., 2019). To sum up, lncRNA GAS5 competitively bound to miR-23a to upregulate PTEN expression. Furthermore, MODE-K cells poorly expressing GAS5 were transfected with miR-23a inhibitor. After the combined treatment of sh-GAS5 + in-miR-23a, the invasion rate of bacteria into MODE-K cells was decreased, and the levels of inflammatory factors were decreased significantly. Reduction in miR-23a markedly inhibits the levels of TNF-α and IL-6 in lipopolysaccharide-induced macrophages (Peng et al., 2015). Inhibition of miR-23a results in increases in IL-12 levels (Du et al., 2018). Briefly, low expression of miR-23a reversed the promoting effect of GAS5 knockdown on *ST* invasion to intestinal epithelial cells.

### p38 MAPK Pathway and Autophagy in CGA Benefit in *ST* Infection

MAPK pathway is a feature of innate immune responses in in *Caenorhabditis elegans* innate immunity (Kim et al., 2002). To explore the downstream pathways regulated by PTEN, Western blot was utilized to detect the expression of p38 MAPK pathway-related proteins. CGA reduced the activation level of p38 MAPK pathway, and knocking down GAS5 partially reversed the inhibitory effect of CGA on p38 MAPK pathway. CGA can block lipopolysaccharide-triggered pro-inflammatory cascades possibly through inhibition of p38 MAPK (Chauhan et al., 2012). CGA attenuates dextran sodium sulfate-induced ulcerative colitis, colonic mucosal damage, inflammation in mice by inactivating the MAPK/ERK/JNK pathway (Gao et al., 2019). When bacteria invade epithelial cells, autophagy is initiated as an autonomous antibacterial defense to prevent intestinal bacterial transmission during *ST* infection (Benjamin et al., 2013). With increasing CGA concentrations, the autophagic vesicles were increased, and LC3II/LC3I was increased, and P62 gradually was decreased. It can be seen that CGA treatment promoted the autophagy of intestinal epithelial cells. To confirm that CGA regulates the autophagy through the lncRNA GAS5/miR-23a/PTEN axis, we performed an *in vitro* functional rescue experiment. Beyond that, after treatment with CGA + sh-PTEN, the autophagy of *ST*-infected MODE-K cells was inhibited, and the invasion rate of *ST* into MODE-K cells increased significantly. In *Caenorhabditis elegans*, CGA improves resistance to thermal via autophagy activation (Carranza et al., 2020). PTEN regulates activation of autophagy in infected macrophages during tularemic infection (Hrstka et al., 2007). Overall, PTEN knockdown inhibited autophagy and reverses the protective effect of CGA against *ST* infection.

### Study Limitation

As we mentioned in the results section, 10 lncRNAs related to immune and anti-inflammatory were found and expressed in the intestinal tissues of mice infected with *ST*. Except for lncRNA GAS5, the others may also have some relevance to the process of *ST* infection. In the future, we will make further investigation to identify the possible mechanisms of other 9 lncRNAs in *ST* infection.

## Conclusion

Overall, our study highlighted the protective effects of CGA on *ST* infection by promoting autophagy in *ST* infection via the GAS5/miR-23a/PTEN axis and p38MAPK pathway.

## Data Availability Statement

The original contributions presented in the study are included in the article/supplementary material, further inquiries can be directed to the corresponding authors.

## Ethics Statement

The animal study was reviewed and approved by the Yunnan University.

## Author Contributions

ST was the guarantor of integrity of the entire study and contributed to the study concepts and definition of intellectual content. FY contributed to the design of this study and manuscript review. QL contributed to the literature research and contributed to the manuscript preparation. YL contributed to the clinical studies. JY took charge of the experimental studies. RL contributed to the data acquisition. ML took charge of the data analysis. ZL contributed to the statistical analysis. FH contributed to the manuscript editing. All authors read and approved the final manuscript.

## Conflict of Interest

The authors declare that the research was conducted in the absence of any commercial or financial relationships that could be construed as a potential conflict of interest.

## Abbreviations

CGA: chlorogenic acid
DMEM: Dulbecco’s modified Eagle’s medium
TSB: tryptic soy broth
EP: eppendorf
PBS: phosphate buffer saline
SPF: specific pathogen free
TBST: tris-buffered saline with Tween
FISH: fluorescence *in situ* hybridization
DAPI: 4 ′, 6-diamidino-2-phenylindole
RT-qPCR: reverse transcription quantitative polymerase chain reaction
TEM: transmission electron microscope
MDC: monodansylcadaverin
PVDF: polyvinylidene fluoride
BCA: bicinchoninic acid
LDH: lactate dehydrogenase
HE: hematoxylin and eosin
ELISA: enzyme-linked immunosorbent assay
MOI: multiplicity of infection
WT: wild type
MUT: mutant
SDS-PAGE: sodium dodecyl sulfate–polyacrylamide gel electrophoresis
BSA: bovine serum albumin
ANOVA: analysis of variance
NO: nitric oxide
TNF- α: tumor necrosis factor- α
IL: interleukin
miR: microRNA
ceRNA: competing endogenous RNA
lncRNA: long non-coding RNAs.
